# Practices and insights from chikungunya prevention and Control in Guangdong, China, 2025

**DOI:** 10.1186/s41182-025-00837-y

**Published:** 2025-11-12

**Authors:** Hengliang Lv, Chunlin Zhou, Yu Chen, Xingshu Chen

**Affiliations:** Department of Military Medical Geography, Army Medical Service Training Base, Army Medical University, Chongqing, China

**Keywords:** Chikungunya, Public health measures, Mosquito-borne disease prevention

## Abstract

Chikungunya, a mosquito-borne disease, has become a global public health concern. In 2025, an imported chikungunya case was detected in Foshan, Guangdong, followed by local spread. This study analyzed the spatiotemporal evolution of this outbreak using data from official channels. Measures like enhanced mosquito control, expanded nucleic acid testing, and activation of emergency response were implemented. As a result, the epidemic was effectively controlled, with no severe cases reported. This work provided valuable insights into chikungunya prevention and control strategies, highlighting the importance of a full-chain prevention–emergency-treatment system for future global efforts.

Chikungunya is an arboviral disease caused by the Chikungunya virus (CHIKV), primarily transmitted by *Aedes aegypti* and *Aedes albopictus*. Clinically, it is characterized by sudden onset of fever, often accompanied by severe joint pain that can be disabling [1, 2]; severe cases may also present with other complications, which further exacerbate harm to the human body. Although two CHIKV vaccines have been approved by regulatory authorities and deployed in some high-risk countries, widespread availability and universal vaccination have not yet been achieved [3]. For most countries worldwide, the prevention and control of chikungunya still rely mainly on macro-epidemiological measures [4]. However, in recent years, global warming has created more favorable conditions for the survival of mosquito-borne pathogens [5]. Therefore, the implementation of effective control strategies is crucial for curbing the transmission of this disease.

The CHIKV was first isolated in Tanzania in 1952, after which it gradually spread to other regions of Africa and Asia. Urban outbreaks occurred in Thailand in 1967 and in India during the 1970s. Since 2004, there has been a significant expansion in the frequency of outbreaks and geographical distribution of the virus in tropical and subtropical regions [1]. As of 2025, CHIKV infection cases have been reported in 119 countries across Asia, Africa, Europe, and the Americas [1, 4]. Previous studies have shown that between 2011 and 2020, 110 countries reported a total of 18.7 million chikungunya cases, resulting in 1.95 million disability-adjusted life years and substantial economic burdens [6].

The prevalence of CHIKV in China dates back to an early period. As early as the 1980s, the viral strain was isolated in Yunnan Province [7]. In recent years, affected by multiple factors such as frequent population mobility and climate change, the number of imported chikungunya cases in China has increased, mainly in southern provinces including Guangdong, Yunnan, and Hainan [2]. On July 8, 2025, a case of imported chikungunya fever was detected through surveillance in Shunde District, Foshan City, Guangdong Province. Subsequently, the epidemic spread to other regions within the province. Health authorities at all levels in China responded promptly and implemented comprehensive public health measures. According to official notification [8], as of August 2, 2025, the rapid upward trend of the chikungunya epidemic in Guangdong Province has been initially contained. This study takes this epidemic as the research object, analyzes the spatiotemporal evolution trend of its distribution, systematically explores China's mitigation strategies and implementation effects for this outbreak, and provides a reference for chikungunya prevention and control in other regions worldwide.

This study collected data on daily confirmed cases, cumulative confirmed cases, and regional distribution of chikungunya in Guangdong Province from July 8 to August 3, 2025, via official channels including the Guangdong Provincial Center for Disease Control and Prevention (https://cdcp.gd.gov.cn/) and Xinhua News Agency (https://www.news.cn/xinhuashe/). Descriptive methods were employed to characterize the temporal and spatial distribution of chikungunya fever in Guangdong Province. Joinpoint regression (version 4.9.1.0; Statistical Methodology and Applications Branch, Surveillance Research Program, National Cancer Institute, Bethesda, MD, USA) was used to identify the temporal trends in CHIKV incidence. The daily percentage change (DPC) was adopted to analyze these trends. A positive and statistically significant DPC indicated an upward trend, whereas a negative DPC indicated a downward trend. If neither condition was met, the trend was considered stable. Furthermore, by integrating the prevention and control strategies implemented in Guangdong Province, an assessment was conducted to evaluate the effectiveness of these strategies. As shown in Fig. 1, the epidemic in Guangdong was divided into two phases: Phase 1 involved the first locally transmitted outbreak in Foshan, triggered by an imported case, with subsequent spread within Foshan (Fig. 1A, B); Phase 2 saw spread from Foshan to other regions in Guangdong (Fig. 1C, D). As shown in Fig. 2, the number of newly reported chikungunya fever cases in Guangdong Province exhibited an upward trend from July 10 to July 18 (DPC = 95.03%, *P* < 0.001), followed by a downward trend from July 18 to August 2 (DPC = − 3.85%, *P* = 0.007).

**Fig. 1:**
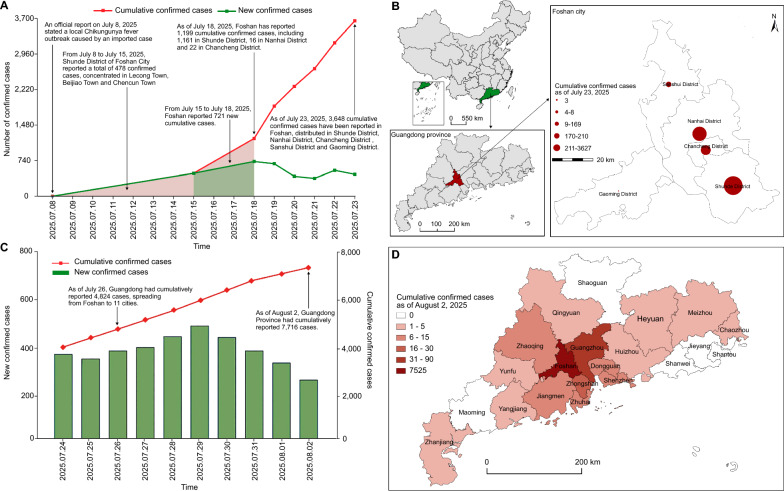
Temporal and spatial trends of chikungunya fever in Guangdong Province, China. Note: **A** Temporal trend of reported chikungunya cases in Foshan City; **B** Spatial distribution characteristics of chikungunya in Foshan City; **C** Temporal trend of reported chikungunya cases in Guangdong Province; **D** Spatial distribution characteristics of chikungunya in Guangdong Province.

**Fig. 2:**
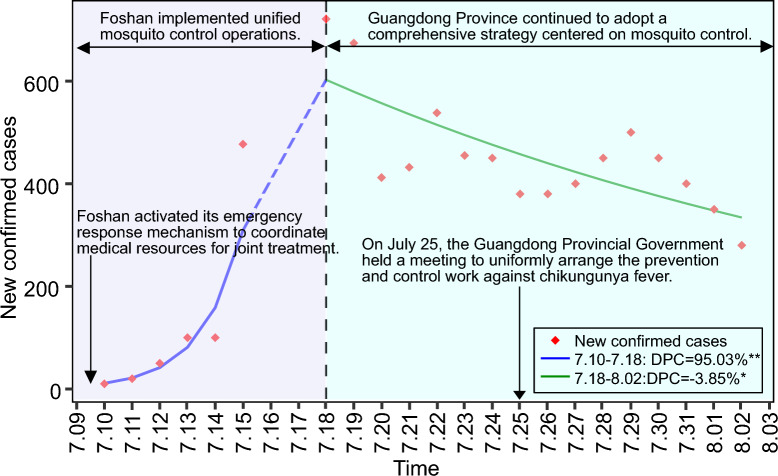
Trend of newly reported chikungunya fever cases in Guangdong Province. Note: DPC: daily percentage change; the dashed line indicated no official reported data between July 15 and July 18. *indicates *p* < 0.05, ** indicates *p* < 0.001

On July 8, 2025, the first imported chikungunya case was officially reported (Fig. 1A). As a coastal city in southern China, Foshan has frequent economic, trade, and population exchanges with overseas countries. The overlapping endemic areas of chikungunya with dengue and Zika in tropical regions—a key geographical and epidemiological feature—confers a high risk of importation [3]. China strengthened health declaration verification for inbound travelers from endemic countries and implemented sanitary treatment for their transport equipment, goods, and parcels to prevent imported cases. Additionally, the Guangdong government mandated enhanced surveillance for early detection of imported and local sporadic cases, coordinated medical resources for treatment, and promoted concurrent prevention of dengue and other diseases. Post-outbreak, Foshan added 35 hospitals for CHIKV nucleic acid testing, stocked sufficient reagents, trained medical staff, and achieved early detection of virus carriers through screening. For case management, Foshan activated an emergency response, designated 53 hospitals as treatment centers, prepared over 3600 mosquito-proof isolation beds, and planned for expansion (Fig. 2). Historically, Guangdong Province has established a multi-sectoral collaboration mechanism and implemented comprehensive prevention and control measures in the prevention and control of mosquito-borne infectious diseases, with relevant effectiveness reflected in the prevention and control of specific diseases. Taking malaria prevention and control as an example, Guangzhou has reported no local malaria cases since 2009, successfully passed the malaria elimination assessment in 2017, and formally achieved the goal of malaria elimination [9].

By July 18, 2025, daily new cases in Foshan had risen continuously, peaking at 721, followed by a downward trend (Fig. 2). As of July 23, 2025, 3,648 cumulative cases were reported across all districts of Foshan: 3,317 in Shunde, 178 in Nanhai, 147 in Chancheng, 6 in Sanshui, and 3 in Gaoming (Fig. 1B). Virus transmission to hosts occurred via virus-carrying mosquitoes. To block transmission, Foshan implemented unified mosquito control: cleaning breeding sites (e.g., back streets, garbage stations), conducting mosquito eradication in crowded areas (schools, markets), and mobilizing residents to clear stagnant water. Professional teams sprayed adult mosquitoes in external environments and key residential areas during peak activity and distributed indoor anti-mosquito products (sprays, coils) to residents.

From July 23 onward, the epidemic remained low. By July 26, 2025, Guangdong had 4824 cumulative cases, spreading to 11 cities including Guangzhou and Zhongshan (Fig. 1C). By August 2, 2025, new cases in Guangdong declined—particularly in Shunde (the core area)—with the rapid upward trend initially curbed [8]. Cumulative cases reached 7716, spreading from Foshan to 16 cities in Guangdong (Fig. 1D). All treated patients were mild, with no severe cases reported after timely treatment. Guangdong adopted a coordinated strategy centered on mosquito control: organizing mass mosquito eradication; deploying specialized equipment to trap Aedes via human odor simulation; and using *Toxorhynchites splendens* and mosquito fish for biological control of Aedes larvae. These methods have demonstrated preliminary effectiveness. For instance, in Guangzhou, surveillance data showed that the Breteau Index decreased from 5.45 in the third week of July to 1.32 in the first week of August. In 2016, Guangdong Province also effectively controlled the travel-transmitted Zika virus through vector control measures [10].

This study also has the following limitations: first, the case data were obtained from official announcements by health authorities, so there may be a lag in the confirmation of the onset time of cases; second, it was not feasible to investigate all policies and measures implemented in Guangdong Province, though the key prevention and control measures have been analyzed; and finally, this manuscript only included population-level data without involving analysis at the genetic level, which requires further expansion in future research.

In summary, the public health measures of chikungunya outbreaks should build a full-chain system of “prevention-emergency-treatment”: in the prevention stage, it is necessary to focus on blocking the source of infection, strengthening the control of overseas input in high-risk areas and periods with high input risks, and reducing the risk of epidemic input from the source; after the outbreak occurs, a unified prevention and control mechanism should be activated, and the spread of the epidemic should be curbed by strengthening monitoring, early warning, and rapid response; at the same time, it is necessary to coordinate and allocate health resources, realize the coordinated promotion of prevention and control measures and clinical treatment, avoid the conversion of mild cases to severe cases, and form a closed-loop management model of “prevention first, orderly emergency response, and combination of prevention and treatment”.

## Data Availability

All data is freely available online.
